# FK866 Protects Human Dental Pulp Cells against Oxidative Stress-Induced Cellular Senescence

**DOI:** 10.3390/antiox10020271

**Published:** 2021-02-10

**Authors:** Chang Youp Ok, Sera Park, Hye-Ock Jang, Takashi Takata, Ok-Hee Lee, Moon-Kyoung Bae, Soo-Kyung Bae

**Affiliations:** 1 Department of Dental Pharmacology, School of Dentistry, Education and Research Team for Life Science on Dentistry, Pusan National University, Yangsan 50612, Korea; luriel@hanmail.net (C.Y.O.); sera8020@naver.com (S.P.); jho9612@pusan.ac.kr (H.-O.J.); 2 Periodontal Disease Signaling Network Research Center, School of Dentistry, Pusan National University, Yangsan 50612, Korea; mkbae@pusan.ac.kr; 3 Tokuyama University, Shunan, Yamaguchi 745-8566, Japan; ttakata@tokuyama-u.ac.jp; 4 Department of Biomedical Science, CHA University, Gyeonggi-do 13488, Korea; okhlee@cha.ac.kr; 5 Department of Oral Physiology, School of Dentistry, Education and Research Team for Life Science on Dentistry, Pusan National University, Yangsan 50612, Korea; 6 Dental and Life Science Institute, School of Dentistry, Pusan National University, Yangsan 50612, Korea

**Keywords:** hydrogen peroxide, senescence, human dental pulp cells, reactive oxygen species, telomere damage, inflammation, SASP factors, NF-κB

## Abstract

FK866 possesses various functional properties, such as anti-angiogenic, anti-cancer, and anti-inflammatory activities. We previously demonstrated that premature senescence of human dental pulp cells (hDPCs) was induced by hydrogen peroxide (H_2_O_2_). The present study aimed to investigate whether H_2_O_2_-induced premature senescence of hDPCs is affected by treatment with FK866. We found that FK866 markedly inhibited the senescent characteristics of hDPCs after exposure to H_2_O_2_, as revealed by an increase in the number of senescence-associated β-galactosidase (SA-β-gal)-positive hDPCs and the upregulation of the p21 and p53 proteins, which acts as molecular indicators of cellular senescence. Moreover, the stimulatory effects of H_2_O_2_ on cellular senescence are associated with oxidative stress induction, such as excessive ROS production and NADPH consumption, telomere DNA damage induction, and upregulation of senescence-associated secretory phenotype factors (IL-1β, IL-6, IL-8, COX-2, and TNF-α) as well as NF-κB activation, which were all blocked by FK866. Thus, FK866 might antagonize H_2_O_2_-induced premature senescence of hDPCs, acting as a potential therapeutic antioxidant by attenuating oxidative stress-induced pathologies in dental pulp, including inflammation and cellular senescence.

## 1. Introduction

Dental pulp is a soft tissue comprising nerves, blood vessels, lymphatic vessels, connective tissue, and various types of cells within a tooth [1]. Moreover, it includes several antibacterial substances, hormones, and immune cells that protect the teeth from external invasive bacteria or irritants and prevent the accumulation of extrinsic bacteria or substances inside the dental pulp [2,3]. Aging and stress-related conditions such as inflammation cause dental pulp cell senescence, leading to the aging of the pulp tissue and impaired tooth protection [4,5,6]. Therefore, it is important to understand the molecular mechanisms that underlie the senescence process of dental pulp cells, in order to maintain healthy teeth.

Cellular senescence is a phenomenon of irreversible cell cycle arrest that limits the replicative life span of cells, which might contribute to age-associated organ diseases [7,8]. Most cells undergo normal cellular senescence with increasing age, while premature cellular senescence can be acutely triggered by various stressors, including ionizing radiation, hyperglycemia, and oxidative stress [9,10,11]. Oxidative stress is a damaging response and refers to excessive intracellular levels of reactive oxygen species (ROS) [12]. Previous studies reported that excess ROS can cause damage to vital biomolecules, such as proteins, lipids, and DNA; moreover, excessive molecular damage in cells might lead to cellular senescence [13]. Since hydrogen peroxide (H_2_O_2_) induces oxidative stress and causes premature senescence in various cell types [14], the in vitro model of premature cellular senescence induced by application of H_2_O_2_ has been used as an efficient tool for studying human aging.

One of the hallmarks of cellular senescence is an acquisition of senescence-associated secretory phenotype (SASP) [15,16]. The SASP consists of a range of secreted factors, such as inflammatory cytokines, growth factors, chemokines, and enzymes that act in auto/paracrine manners [17]. Short-term acute induction of the SASP can prove to be beneficial for tissue regeneration and tumor suppression through the clearance of dysfunctional cells, whereas long-term chronic exposure to SASP factors in aging or lesions facilitates inflammatory, tumorigenic, and age-associated diseases [18]. The expression of key SASP genes is transcriptionally regulated by the NF-κB transcription factor in response to senescence inducers [19,20]. Upon exposure to the inducers, the degradation of the inhibitors of NF-κB (IkBs), following phosphorylation, leads to the NF-κB activation and nuclear translocation, affecting the transcription of the NF-κB-dependent SASP genes [19,20,21].

We recently reported that visfatin induced senescence in human dental pulp cells (hDPCs), which was inhibited by FK866, a noncompetitive small-molecule inhibitor of visfatin [22]; however, the role of FK866 in H_2_O_2_-induced cellular senescence remains unknown. Therefore, the present study was conducted to evaluate whether FK866 protects hDPCs from premature senescence induced by H_2_O_2_. To achieve this, we assessed the in vitro effects of H_2_O_2_ on hDPCs with regard to the expression of a series of senescence markers, including the senescence-associated β-galactosidase, and the expression of the p21 and p53 proteins. We further aimed to reveal the mechanism through which FK866 affects H_2_O_2_-induced senescence in hDPCs via molecular analyses, such as ROS production, NADPH consumption, telomere dysfunction, and SASP genes expression.

## 2. Materials and Methods

### 2.1. Reagents

Hydrogen peroxide (H_2_O_2_) was obtained from Junsei (Tokyo, Japan). FK866 was purchased from Adipogen (Switzerland). The antibodies used in the present study were as follows—rabbit anti-p21 (Santa Cruz Biotech, Dallas, TX, USA), mouse monoclonal anti-p53 (Calbiochem, San Diego, CA, USA), mouse monoclonal anti-β-Actin (Abcam, Cambridge, MA, USA), mouse monoclonal anti-TRF1 (Santa Cruz Biotechnology, Dallas, TX, USA), rabbit anti-phospho (Ser139)-histone H2AX (γH2AX) (Cell Signaling Technology, Danvers, MA, USA), mouse monoclonal anti-NF-κB p65 (Santa Cruz Biotech, Dallas, TX, USA), horseradish peroxidase-conjugated goat anti-rabbit, and anti-mouse IgG (Thermo Fisher Scientific, Waltham, MA, USA), Alexa Fluor^®^ 488-conjugated goat anti-mouse IgG, and Alexa Fluor^®^ 594-conjugated goat anti-rabbit IgG (Invitrogen, Camarillo, CA, USA).

### 2.2. Cell Culture

A cell line of immortalized human dental pulp cells (hDPCs) [23] were cultured in Dulbecco’s modified Eagle’s medium (DMEM), supplemented with 10% heat-inactivated fetal bovine serum (FBS, GibcoTM, Gaithersburg, MD, USA) and 1% antibiotics–antimycotics (GibcoTM, USA). Primary human umbilical vein endothelial cells (HUVECs) were purchased from CLONETICS (Lonza, Bazel, Switzerland). HUVECs were plated on 0.2% gelatin-coated plate and cultured in sterile endothelial growth medium with trace element and growth factors. Murine bone marrow-derived macrophages (mBMDM) out of the femur and tibia of hind limbs of 6- to 8-week old mice were generated by isolating the bone marrow. Cells were differentiated directly in 100 mm culture dish in the presence of 20 ng/mL macrophage colony-stimulating factor (M-CSF) (Peprotech, Hamburg, Germany) for up to 7 days. At day 3, fresh M-CSF was added. Human dental pulp stem cells (hDPSCs) were purchased from Lonza Inc. (PT-5025, Walkersville, MD, USA). The hDPSCs were cultured in StemMACS^TM^ MSC Expansion media Kit XF (130-104-182, Miltenyi Biotec., Inc., Somerville, MA, USA) with 20% FBS and 1% antibiotics–antimycotics. The cells were incubated at 37 °C in a humidified 5% CO_2_ atmosphere.

### 2.3. Reverse Transcriptase Polymerase Chain Reaction (RT-PCR)

Total RNA was isolated from hDPCs using a TRIzol reagent kit (Invitrogen, Carlsbad, CA, USA). cDNA synthesis was performed using 1 μg of total RNA with MaximeRT premix (iNtRON Biotechnology, Sungnam, South Korea). The oligonucleotide primer sequences used for PCR were as Table 1.

### 2.4. Western Blot Analysis

The harvested cells were lysed in radioimmunoprecipitation assay buffer (RIPA) (iNtRON Biotechnology, Sungnam, Korea) containing a protease inhibitor cocktail (Roche, Mannheim, Germany). Protein extracts (30 μg/lane) were separated by SDS-PAGE and transferred to a nitrocellulose membrane (Amersham Pharmacia Biotech, Little Chalfont, UK). The membrane was blocked with 5% skim milk in PBS containing 0.1% Tween 20 for 1 h at room temperature and probed with the appropriate antibodies. The signal was developed using an ECL detection system (Amersham Pharmacia Biotech, Little Chalfont, UK).

### 2.5. SA-β-galactosidase Staining Assay

The degree of SA-β-galactosidase activity was measured using a senescence assay kit (Senescence Cells Histochemical Staining; Sigma-Aldrich, St. Louis, MO, USA), according to the manufacturer’s protocol. The cells were washed twice in 1× PBS and fixed in 1× fixation buffer for 6–7 min at 25 °C, washed in 1× PBS, and incubated overnight with SA-β-galactosidase staining solution at 37 °C without CO_2_. Thereafter, the cells were imaged under a microscope (Olympus, IX71; Toyko, Japan).

### 2.6. ROS Measurement

The ROS-ID^®^ Total ROS detection kit (Enzo, Farmingdale, NY, USA) was used to measure the total intracellular ROS generation. For measurement of intracellular ROS levels, the cells (1 × 10^4^ cells) were seeded in a 96-well dark plate overnight and incubated with oxidative stress detection reagent in cell culture media at 37 °C in a dark room for 30 min, as recommended by the manufacturer. Fluorescence was measured at a wavelength of 490 nm (excitation) / 525 nm (emission), using a fluorescent microplate reader (BIOTEK, Cytation3, USA). For ROS detection in cells, the cells (5 × 10^3^ cells) were seeded in a 24-well plate with poly L-lysine-coated coverslips overnight, and incubated with oxidative stress detection reagent in cell culture media at 37 °C in a dark room for 30 min, following the manufacturer’s instructions. After washing with buffer salts thrice, the cells were analyzed using a confocal microscope LSM510 (Carl Zeiss, Oberkochen, Germany).

### 2.7. NADP/NADPH Assay

The NADP/NADPH levels were assessed using a colorimetric NADP/NADPH assay kit (Abcam, Cambridge, MA, USA), following the manufacturer’s instructions. The cells were lysed in the assay buffer provided in the kit. The lysates were deproteinized by passing through a 10-kD spin column (Biovision, Milpitas, CA, USA). Thereafter, the assay was performed in a 24-well plate, and the absorbance of the samples was measured using a Multimode Plate Reader Victor X3, P (Perkin Elmer, Hopkinton, MA, USA) at 450 nm.

### 2.8. Immunocytochemistry

Cells cultured on poly L-lysine-coated coverslips were washed thrice with phosphate-buffered saline (PBS), fixed in 4% paraformaldehyde/PBS for 10 min at room temperature, permeabilized with 0.01% Triton X-100 in PBS for 15 min at room temperature, and then washed thrice with PBS for 5 min. Thereafter, the cells were blocked with 1% BSA/0.3% Triton X-100/PBS for 1 h and labeled with the appropriate primary antibodies. After overnight incubation at 4 °C, the cells were incubated with Alexa Fluor 488-conjugated secondary antibodies for 1 h at 25 °C. Coverslips were mounted on slides with a fluorescent mounting medium containing DAPI (Vector Laboratories, Burlingame, CA, USA). Images were acquired using a confocal microscope (LSM 510; Carl Zeiss; Oberkochen, Germany).

### 2.9. Statistical Analysis

Data are presented as the mean ± standard deviation (SD) obtained from at least three independent experiments. Statistical analysis was performed using Student’s *t*-test for data points and ANOVA for curves.

## 3. Results

### 3.1. FK866 Alleviates H_2_O_2_-Induced Premature Senescence in hDPCs

**Figure 1:**
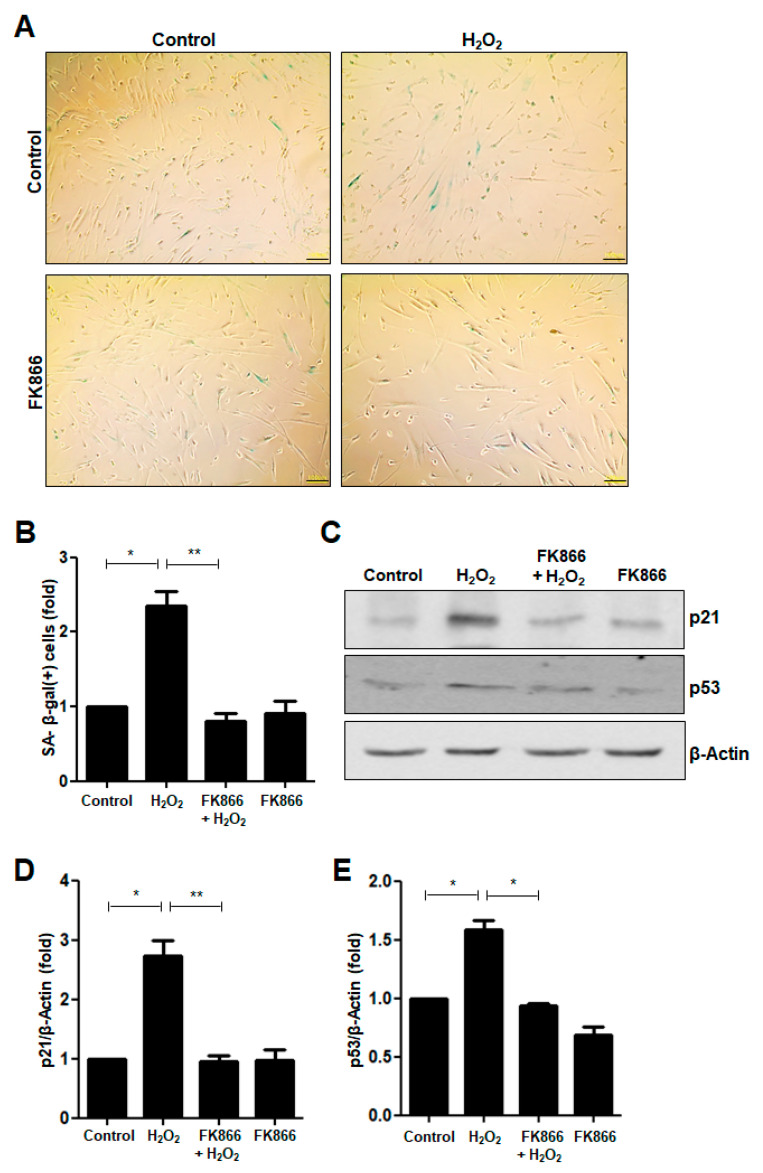
Effect of FK866 on H_2_O_2_-induced premature senescence in hDPCs. (**A**) The hDPCs were stimulated with H_2_O_2_ (400 nM) for 24 h and stained for detecting the activity of senescence-associated (SA)-β-galactosidase. Representative image of SA-β-galactosidase staining. Scale bar: 100 μm. (**B**) Quantitative results for the percentage of cells stained positively with SA-β-galactosidase. (**C**) The hDPCs were pretreated with FK866 (10 µM) for 2 h and then stimulated with H_2_O_2_ (400 nM) for 24 h. Cell extracts were subjected to Western Blot analysis for detecting the levels of the p21 and p53 proteins. β-Actin was used as the loading control. (**D**,**E**) Densitometric analysis for assessing the relative protein levels were normalized to the levels of β-Actin: D, p21; E, p53. * *p* < 0.1, ** *p* < 0.01.

We recently reported that 400 nM H_2_O_2_ did not affect the survival of hDPCs and induced premature senescence of immortalized hDPCs [24]. In this study, we confirmed that H_2_O_2_ increased the fraction of cells that was stained positive for SA-β-galactosidase (SA-β-gal), a marker of cellular senescence (Figure 1A,B). To address the effect of FK866 on H_2_O_2_-induced premature senescence, the hDPCs were pretreated with FK866 before H_2_O_2_ treatment. As shown in Figure 1A,B, the increase in the number of SA-β-gal(+) cells elicited by H_2_O_2_ was reversed by the FK866 pretreatment. We examined whether FK866 affects H_2_O_2_-induced premature senescence in other different cells including vascular endothelial cells and macrophages, and obtained similar results using HUVEC (Figure S1A,B) and mBMDM (Figure S1C,D). In the case of human dental pulp stem cells (hDPSCs), treatment with 400 nM H_2_O_2_ had no significant effect on the induction of cellular senescence (Figure S1E).

The change in the fraction of SA-β-gal(+) cells among the H_2_O_2_-treated hDPCs was evaluated by detecting the expression of the p21 and p53 proteins, which are well-known senescence markers. H_2_O_2_ increased the protein levels of p21 (Figure 1C,D) and p53 (Figure 1C,E) in hDPCs. When pretreated with FK866 before H_2_O_2_ treatment, the H_2_O_2_-induced increase in the levels of p21 and p53 proteins was significantly attenuated (Figure 1C–E).

### 3.2. FK866 Attenuates H_2_O_2_-Induced Telomere Damage in hDPCs

**Figure 2:**
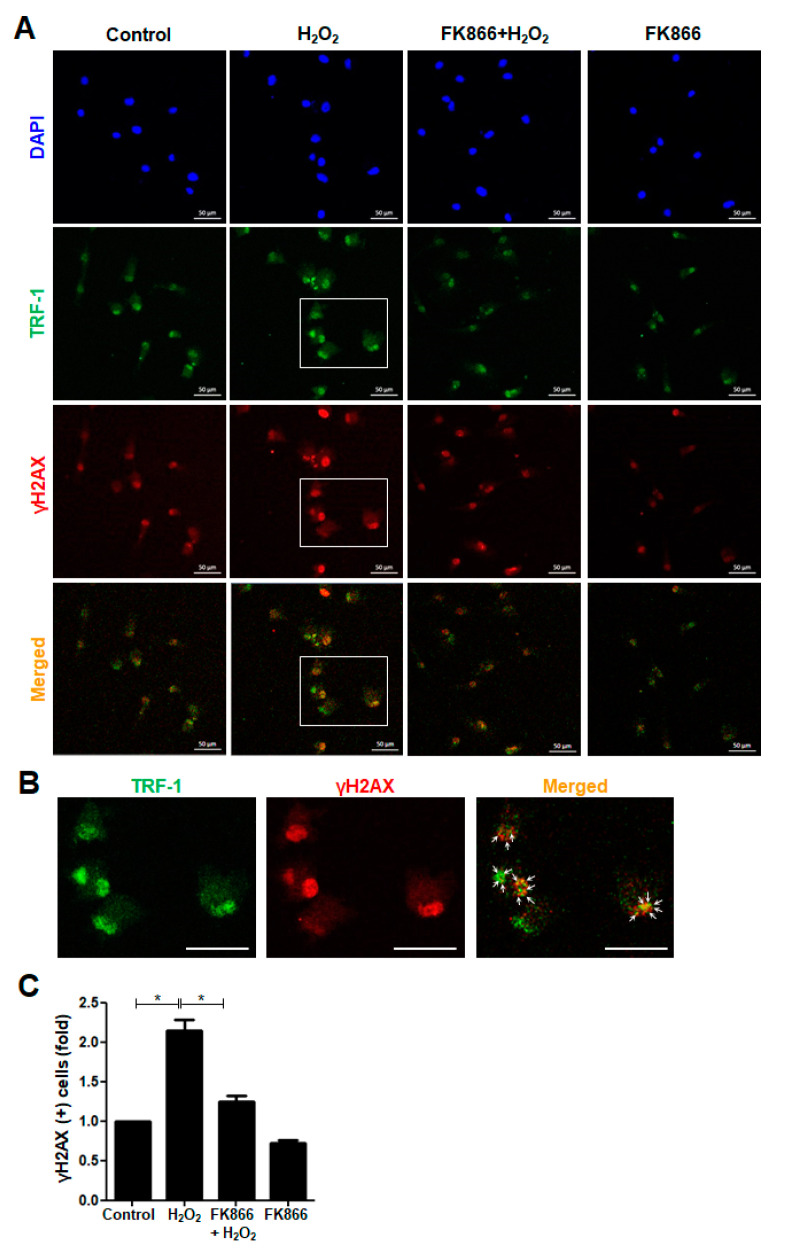
Effect of FK866 on H_2_O_2_-induced telomere damage formation in hDPCs. The hDPCs were pretreated with FK866 (10 µM) for 2 h and then stimulated with H_2_O_2_ (400 nM) for 24 h. (**A**) Immunofluorescence analysis of TRF-1 (Alexa fluoro (AF)-488, green) and γH2AX (AF-594, red) in hDPCs. The cells were analyzed using a confocal microscope. The nuclei were counterstained with DAPI (blue). (**B**) Higher magnification images of the boxed areas (**A**) showing double immunoreactivity (yellow) for TRF-1(green) and γH2AX(red). Arrows in the merged images point to sites of colocalization. Scale bar: 50 μm. (**C**) Quantitative result for the percentage of γH2AX-positive cells. * *p* < 0.1.

H_2_O_2_, the most abundant ROS, causes DNA damage via the promotion of intracellular oxidative stress [14,25]. To examine whether H_2_O_2_ treatment influences the DNA damage response in hDPCs, we performed immunocytochemistry and detected γH2AX foci formed at the sites where the DNA was damaged and at sites with uncapped dysfunctional telomeres. The intensity of γH2AX-positive immunofluorescence staining increased in response to H_2_O_2_ treatment (Figure 2A,B). Moreover, H_2_O_2_ increased the number of γH2AX foci that were scattered over the nucleus and colocalized with telomeric TRF-1 signals (Figure 2A,B). Furthermore, the H_2_O_2_-induced increase in both the intensity and number of γH2AX-positive cells was significantly attenuated by pretreatment with FK866 (Figure 2A,C).

### 3.3. FK866 Reverses H_2_O_2_-Induced Upregulation of NADPH Consumption and ROS Production

**Figure 3:**
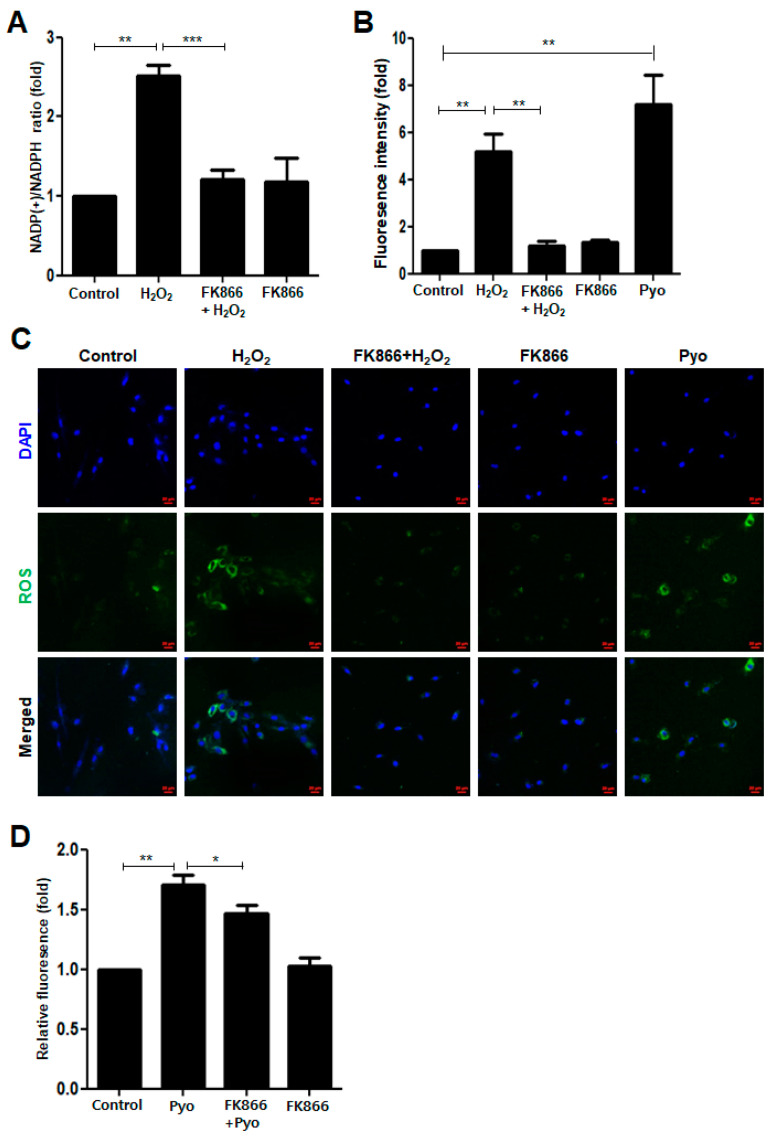
Effect of FK866 on H_2_O_2_-induced NADPH consumption and ROS production in hDPCs. (**A**–**C**) The hDPCs were pretreated with or without FK866 (10 µM) for 2 h and then stimulated with H_2_O_2_ (400 nM) for 24 h or pyocyanin (250 µM) for 30 min. Measurement of the NADP(+)/NADPH ratio in H_2_O_2_-treated hDPCs with or without FK866 pretreatment (**A**). Measurement of the total intracellular ROS levels in H_2_O_2_-treated hDPCs with or without FK866 pretreatment using a fluorescent microplate reader (**B**) and a confocal microscope (**C**). Pyocyanin, a redox-active compound that generates ROS, was used as a positive control for ROS production. The nuclei were counterstained with DAPI (blue). Scale bar: 20 μm. (**D**) The hDPCs were pretreated with FK866 (10 µM) for 2 h and then stimulated with pyocyanin (Pyo) for 30 min. * *p* < 0.1, ** *p* < 0.01, *** *p* < 0.001.

DNA damage is caused by various stresses including oxidative stress [26]. Unless such DNA damage is repaired, cells undergo premature senescence [15,27]. To examine whether oxidative stress is involved in H_2_O_2_-induced senescence in hDPCs, we measured the NADP^+^/NADPH ratio and ROS production, two major indicators of oxidative stress [26,28]. As shown in Figure 3A, H_2_O_2_ increased the NADP^+^/NADPH ratio, which was reversed by the FK866 treatment. In accordance with the changes in the NADP+/NADPH ratio, intracellular ROS levels were remarkably elevated after H_2_O_2_ treatment in hDPCs, whereas FK866 pretreatment reversed the H_2_O_2_-induced increase in ROS levels (Figure 3B). Similar results were obtained with HUVEC (Figure S2A), mBMDM (Figure S2B), and hDPSCs (Figure S2C). Moreover, in the immunofluorescence analysis, the green intensity per cell was higher in the H_2_O_2_-treated hDPCs than in the control cells (Figure 3C). The stimulatory effect of H_2_O_2_ on intracellular ROS production was verified using pyocyanin (Pyo) as a positive “ROS generator” control (Figure 3B,C). Furthermore, FK866 decreased the extent of Pyo- induced ROS production (Figure 3D).

### 3.4. FK866 Reverses H_2_O_2_-Induced Upregulation of SASP Factors

**Figure 4:**
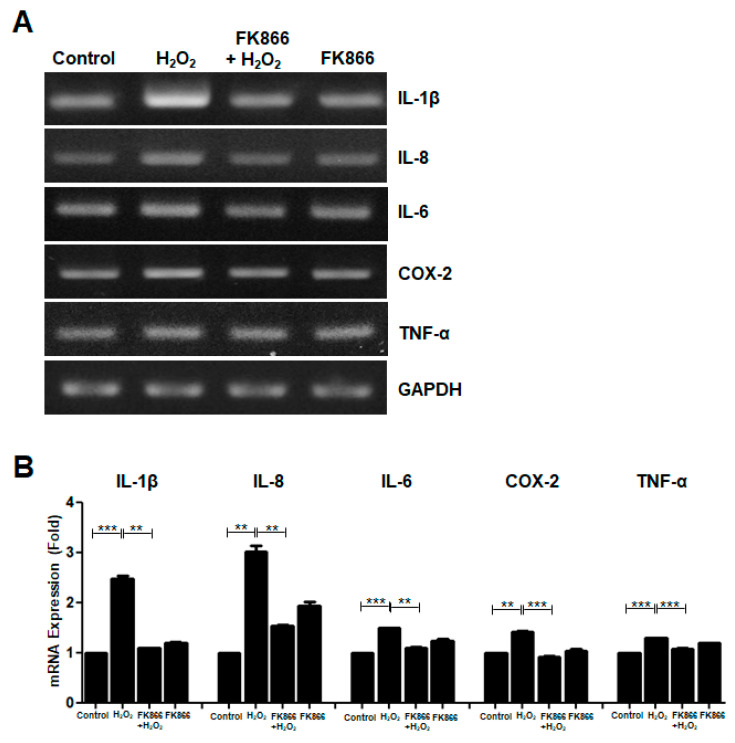
Effect of FK866 on the H_2_O_2_-induced upregulation of SASP factors in hDPCs. The hDPCs were pretreated with FK866 (10 µM) for 2 h and then stimulated with H_2_O_2_ (400 nM) for 24 h. (**A**) The cell lysates were subjected to RT-PCR for determining the mRNA levels of the indicated SASP markers. GAPDH was used as an internal control. (**B**) Densitometric analysis for assessing the relative mRNA expression level of each SASP factor normalized to the mRNA level of GAPDH. ** *p* < 0.01, *** *p* < 0.001.

The SASP is a hallmark of cellular senescence [16]. To investigate whether H_2_O_2_ affects the induction of SASP, the expression levels of several SASP factors were examined by RT-PCR in H_2_O_2_-treated hDPCs. H_2_O_2_ increased the mRNA levels of SASP factors such as IL-1β, IL-8, IL-6, COX-2, and TNF-α, which were all reduced by pretreatment with FK866 in hDPCs (Figure 4A,B).

### 3.5. FK866 Reverses H_2_O_2_-Induced NF-κB Activation

**Figure 5:**
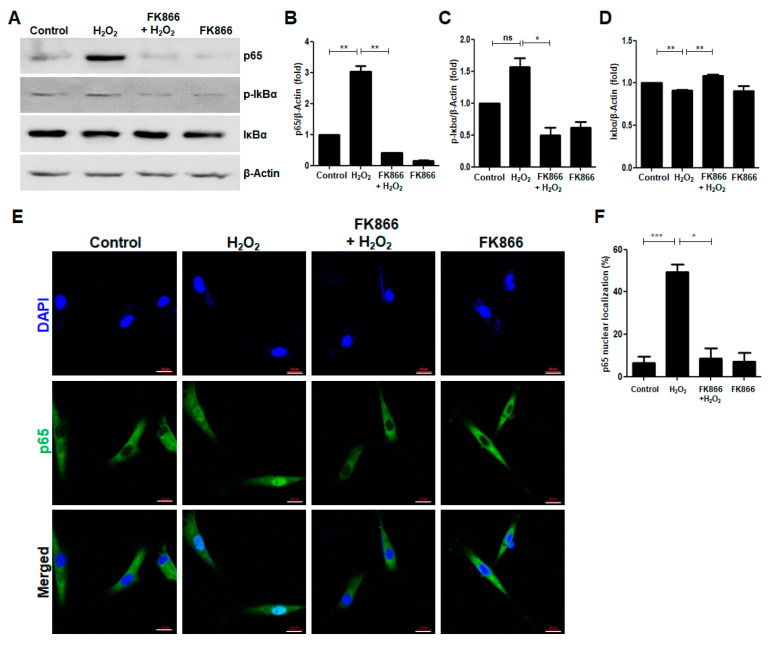
Effect of FK866 on H_2_O_2_-induced upregulation of NF-κB p65 activation in hDPCs. The hDPCs were pretreated with FK866 (10 µM) for 2 h and then stimulated with H_2_O_2_ (400 nM) for 24 h. (**A**) The cell extracts were subjected to Western Blot analysis for detecting the expression of the NF-κB p65, p-IκBα, and IκBα proteins. β-Actin was used as the loading control. (**B**–**D**) Densitometric analysis for assessing relative protein levels normalized to the levels of β-Actin: B, p65; C, p-IκBα; D, IκBα. (**E**) Immunocytochemical analysis of NF-κB p65 protein localization in the H_2_O_2_-treated cells. The cells were visualized using a fluorescence microscope. The nuclei were counterstained with DAPI (blue). (**F**) Quantitative results for the percentage of nuclear localization of NF-κB p65. Scale bar: 20 μm. * *p* < 0.1, ** *p* < 0.01, *** *p* < 0.001.

Since NF-κB is one of main transcription factors involved in the activation of SASP in response to senescence inducers [17,19,20], Western Blotting and immunocytochemistry were performed to examine whether H_2_O_2_ affects NF-κB activation in hDPCs. It was observed that H_2_O_2_ increased the protein levels of the NF-κB p65 and the phosphorylation of Ser-32 in IκBα (p-IκBα), whereas the levels of IκBα protein decreased in H_2_O_2_-treated hDPCs (Figure 5A–C). Furthermore, H_2_O_2_-induced nuclear translocation of the p65 subunit of NF-κB was abrogated by the FK866 pretreatment (Figure 5E,F).

## 4. Discussion

In the present study, we demonstrated the effect of FK866 on the senescence of human dental pulp cells in an in vitro model of H_2_O_2_-induced premature cellular senescence. This was the first study to report that FK866 exerts protective effects against senescence in H_2_O_2_-treated hDPCs. FK866 inhibited H_2_O_2_-induced ROS generation, NADPH consumption, and upregulation of SASP in hDPCs. Furthermore, FK866 prevented H_2_O_2_-induced NF-κB activation in hDPCs.

Oxidative stress is implicated in normal aging and age-associated diseases, including neurodegenerative diseases, cardiovascular diseases, diabetes, and cancers [29,30,31,32,33]. Overproduction of intracellular ROS causes oxidative stress, which majorly contributes to the induction and progression of cellular senescence. Moreover, H_2_O_2_ activates the signaling pathways to promote ROS production and thus, induces premature cellular premature senescence via oxidative stress in various cells, such as human retinal epithelial cells, hippocampal neurons, vascular endothelial cells, and human periodontal ligament cells [25,34,35,36,37]. In previous studies, we reported that H_2_O_2_ caused premature senescence in hDPCs; however, the involvement of oxidative stress in this process was not elucidated [24]. In the present study, we demonstrated that H_2_O_2_ induces senescence in hDPCs by stimulating NADPH consumption and increasing ROS generation, whereas pretreatment with FK866 significantly abrogated this effect, suggesting that FK866 acts as an antioxidant against oxidative stress-induced senescence in hDPCs during H_2_O_2_ treatment. Furthermore, we found that the ROS generator pyocyanin remarkably increased ROS production in hDPCs, whereas FK866 decreased pyocyanin-induced ROS production. Therefore, based on these results, it is conceivable that FK866 prevents H_2_O_2_-induced oxidative stress in hDPCs and further protects the cells against oxidative stress-induced senescence. Thus, it would be interesting to study whether FK866 exerts antioxidant effects on other oxidative stress-related human diseases, such as viral infection, cardiovascular diseases, and oral mucosal diseases [34,35,36,37,38,39,40].

FK866 exhibits anti-inflammatory and antitumor effects [41]; however, its role in cellular senescence is debatable. The results of the present study revealed that FK866 attenuated H_2_O_2_-induced senescence in hDPCs, which was accompanied by a decrease in ROS production. Similar effects of FK866 were observed in vascular endothelial cells (HUVEC) and macrophages (mBMDM). On the other hand, in the case of human dental pulp stem cells (hDPSCs), FK866 exerted inhibitory effects on H_2_O_2_-induced ROS production, but the effect of FK866 on cellular senescence could not be further analyzed due to negative SA-β-gal staining in hDPSCs that were treated with 400 nM H_2_O_2_ for 1 day. It was recently reported that cellular senescence of primary human DPSCs isolated from dental pulp tissues was induced after incubation with 100 µM H_2_O_2_ for 7 days [42]; we therefore consider future research to examine whether culture conditions with much higher concentrations and longer time of H_2_O_2_ treatment could lead to the senescence of hDPSCs and to address if the senescence could be reversed by FK866. Based on these results, it is likely that the protective role of FK866 as an antioxidant against oxidative stress-induced senescence is probably conserved in several cell types, which is in accordance with the results on human endothelial cells [43], but is inconsistent with the stimulatory effects of FK866 on the senescence of other cell types [44,45]. The reason for this discrepancy is unknown; however, this might result from the differences in the cell type- and context-dependent effects of FK866.

In this study, we found that FK866 reversed H_2_O_2_-induced NF-κB p65 activation as well as cellular senescence. Thus, considering growing evidence that the NF-κB activation plays an important role in senescence and aging processes [7,17,20,46,47], it is likely that FK866 inactivated-NF-κB p65 might have an impact on FK866-protection in H_2_O_2_-induced senescence. At present, we do not know whether the protection of FK866 against H_2_O_2_-induced senescence is directly or indirectly attributable to the inactivation of NF-κB p65 in hDPCs. Thus, additional studies are needed to investigate the effects of NF-κB p65 inactivation on FK866-protection in H_2_O_2_-induced senescence as well as on H_2_O_2_-induced senescence, through which the relationship between FK866-protection in H_2_O_2_-induced senescence and NF-κB p65 pathway would be clarified.

Hydrogen peroxide was used in dentistry since 1913 to control periodontal disease and reduce tooth plaque [48]. H_2_O_2_ exhibits antimicrobial activity in the oral cavity by releasing oxygen, destroying the cell wall of bacteria, and subsequently exerting a pathogenic effect against both gram-positive and gram-negative bacteria; thus, it could be used for commercial dentifrices in periodontal therapy [48,49]. A recent study reported that H_2_O_2_ produced by *Streptococcus sanguinis* reduced the biomass of *Porphyromonas gingivalis* [50]. *Aggregatibacter actinomycetemcomitans* protects *P. gingivalis* from H_2_O_2_ attack via catalase-mediated H_2_O_2_ degradation, which consequently aided the survival of anaerobic *P. gingivalis* in *S. sanguinis*-*P. gingivalis*-*A. actinomycetemcomitans* tri-species biofilms under micro-aerobic conditions, indicating the therapeutic role of H_2_O_2_ in treating *P. gingivalis*-mediated periodontal diseases [50]. In addition, H_2_O_2_ is frequently used in dentistry as a tooth bleaching agent due to its oxidative reactivity [48,49]. In addition to these advantages, H_2_O_2_ is widely known as a cytotoxic substance [51]. H_2_O_2_ is produced in vivo by the dismutation of superoxide radicals, and presumably, most human cells are exposed to different levels of H_2_O_2_. Exposure to high concentrations of H_2_O_2_ corrodes tissues, organs, and mucous membranes [49,51]. Sublethal H_2_O_2_ exposure leads to cellular senescence in tissues and organs [52]. Our results demonstrated that sublethal H_2_O_2_ induced premature senescence in hDPCs. Thus, considering that numerous environmental stressors trigger H_2_O_2_ production, the increased use of agents containing or generating H_2_O_2_ in oral hygiene requires continuous monitoring, and safety concerns regarding the senescent effects of H_2_O_2_ on oral tissues and cells exist.

## 5. Conclusions

In conclusion, the present study demonstrated that FK866 attenuates oxidative stress and reverses oxidative stress-induced senescence in hDPCs. Therefore, FK866 might serve as a potential pharmacological agent used to maintain and promote the health of dental pulp tissues.

## Figures and Tables

**Table 1:** The oligonucleotide primer sequences used for PCR.

Gene	Accession Number	Primer Sequence
GAPDH	NM_001256799.3	Forward: 5′-ATCTTCCAGGAGCGAGATCC-3′
		Reverse: 5′-AGGAGGCATTGCTGATGATC-3′
IL-1β	NM_000576.3	Forward: 5′-GGATATGGAGCAACAAGTGG-3′
		Reverse: 5′-ATGTACCAGTTGGGGAACTG-3′
IL-6	NM_000600.5	Forward: 5′-AGATTCCAAAGATGTAGCCG-3′
		Reverse: 5′-TCTTTGCTGCTTTCACACAT-3′
IL-8	NM_000584.4	Forward: 5′-ATGACTTCCAAGCTGGCCGTGGCT-3′
		Reverse: 5′-CTCAGCCCTCTTCAAAAACTTCTC-3′
COX-2	NM_000963.4	Forward: 5′-TTCTTTGCCCAGCACTTCAC-3′
		Reverse: 5′-CTGCTCATCACCCCATTCAC-3′
TNF-α	NM_000594.4	Forward: 5′-GAGTGACAAGCCTGTAGCCCA-3′
		Reverse: 5′-GCAATGATCCCAAAGTAGACC-3′

## Data Availability

All datasets generated for this study are included in the article.

## Supplementary Materials

The following are available online at https://www.mdpi.com/2076-3921/10/2/271/s1. Figure S1. Effect of FK866 on H_2_O_2_-induced premature senescence in HUVEC, mBMDM, and hDPSCs; Figure S2. Effect of FK866 on H_2_O_2_-induced ROS production in HUVEC, mBMDM, and hDPSCs.
