# Supplementary material for: FK866 Protects Human Dental Pulp Cells against Oxidative Stress-Induced Cellular Senescence
Source: Antioxidants (Basel). 2021 Feb 10;10(2):271. doi: 10.3390/antiox10020271 (PMC7916510; doi:10.3390/antiox10020271)
Supplement: Supplementary file 1 [file antioxidants-10-00271-s001.pdf]

antioxidants-1083021 (Round 2)

Supplementary data

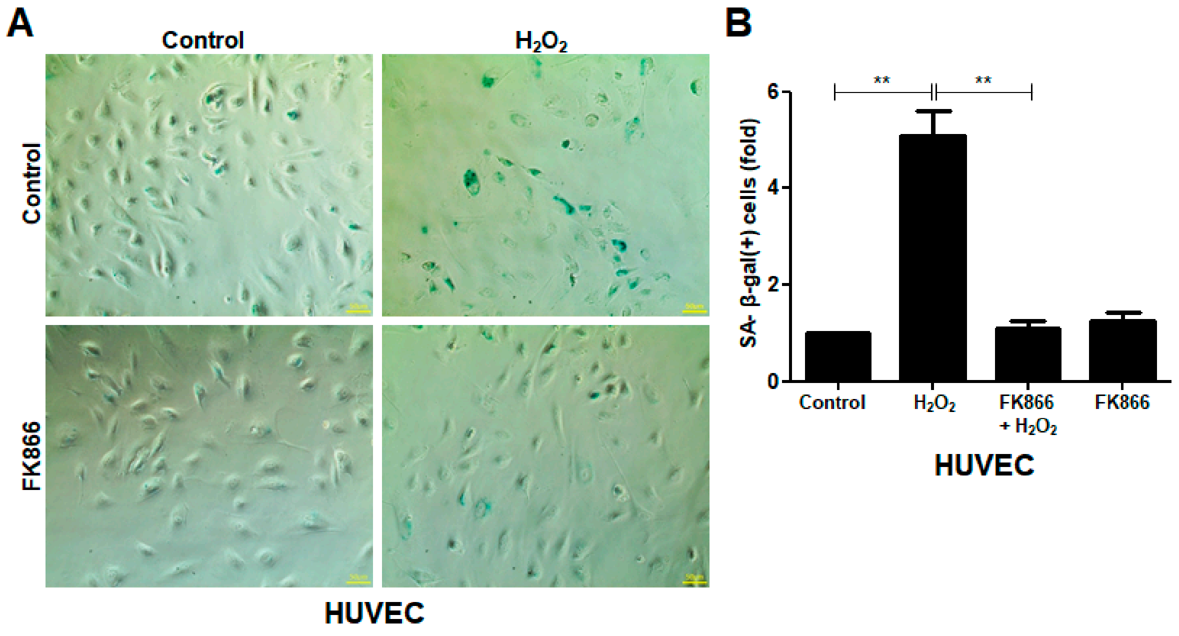

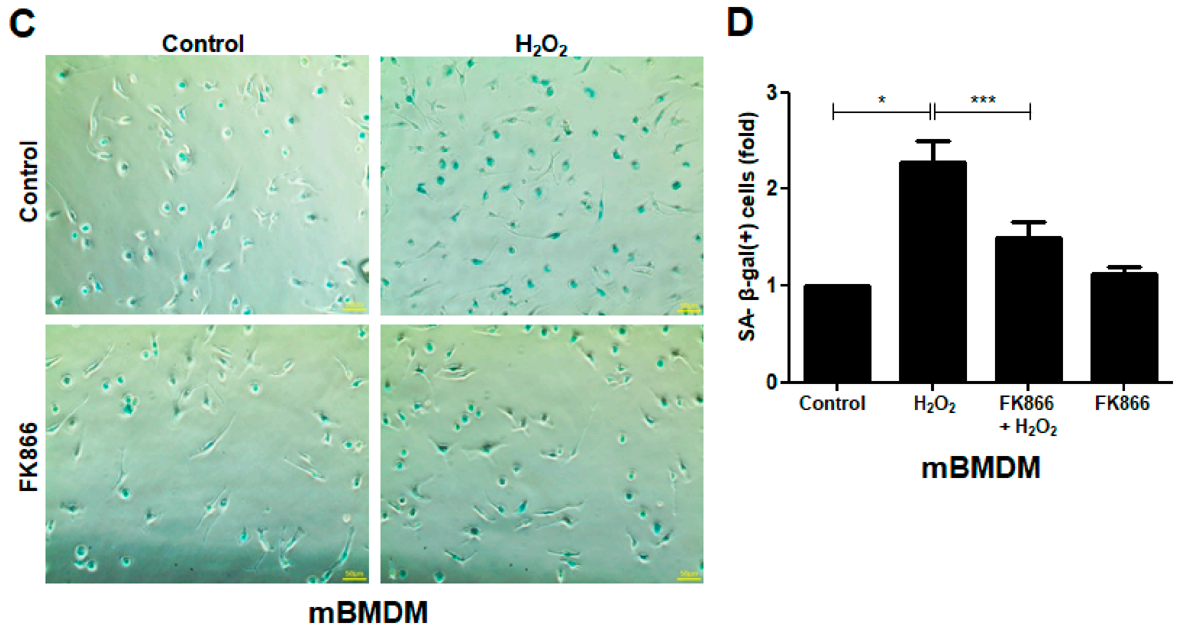

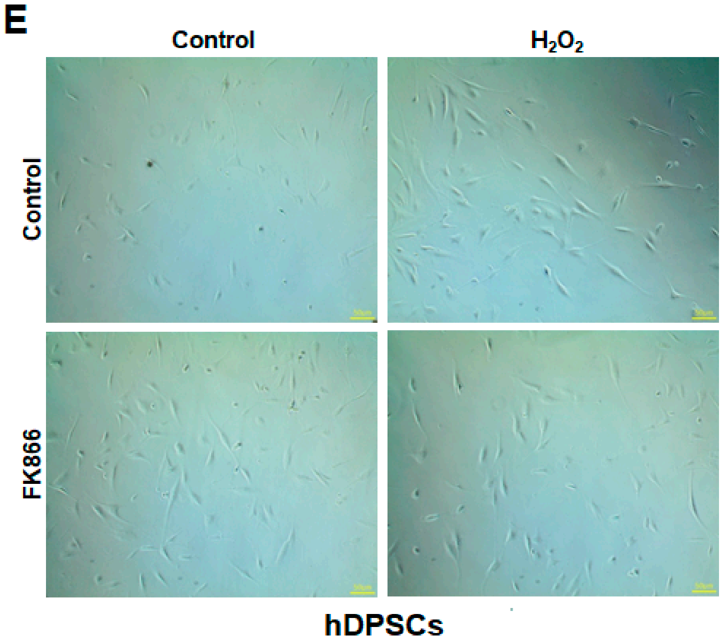

Figure S1. Effect of FK866 on H_2O_2-induced premature senescence in HUVEC (A,B), mBMDM (C,D), and hDPSCs (E). Cells were stimulated with H_2O_2 (400 nM) for 24 h and stained for detecting the activity of senescence-associated (SA)-β-galactosidase. (A,C,E) Representative image of SA-β-galactosidase staining. (B,D) Quantitative results for the percentage of cells stained positively with SA-β-galactosidase. Scale bar: 50 µm. *$p < 0.1$, **$p < 0.01$, ***$p < 0.001$.

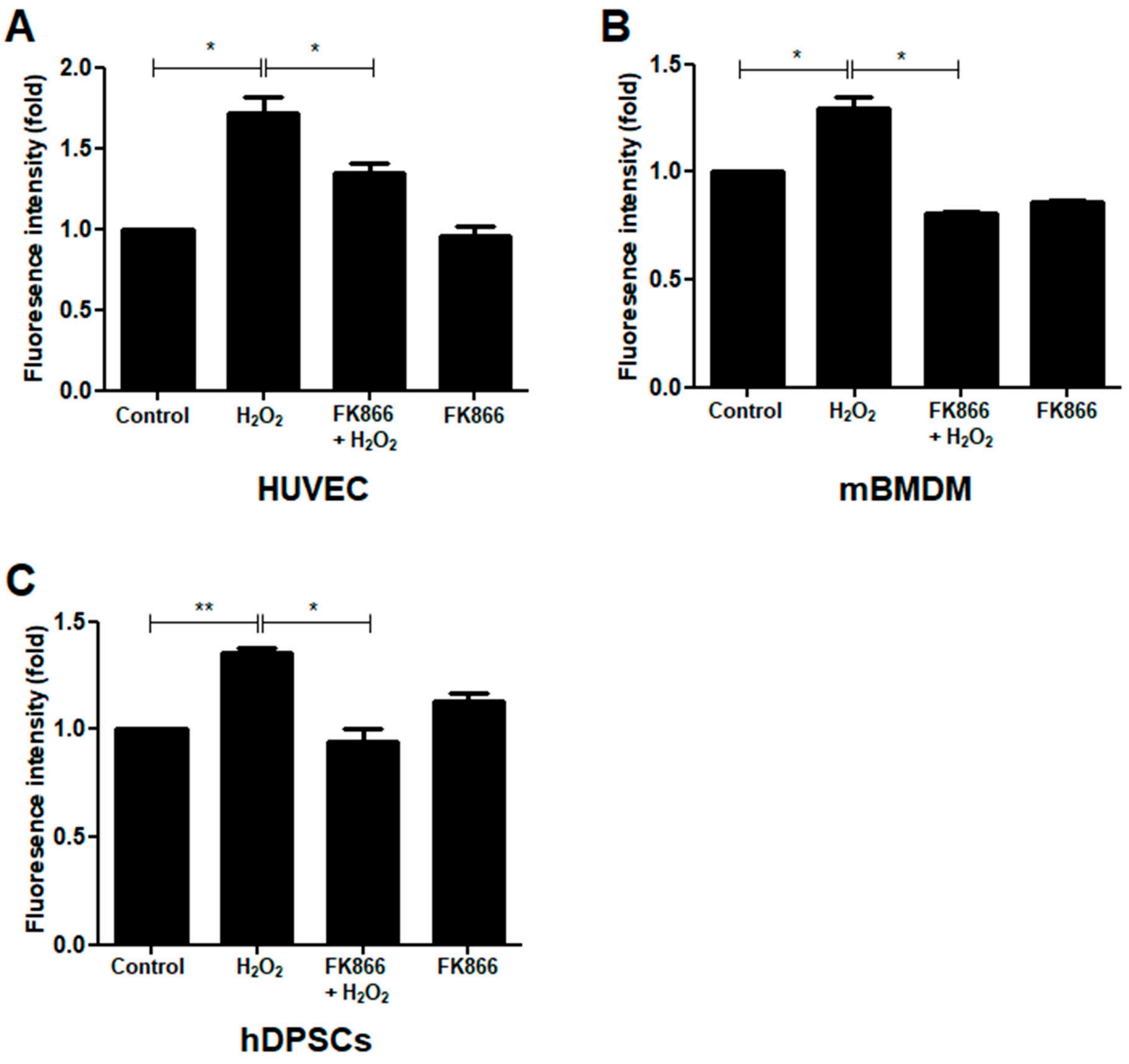

Figure S2. Effect of FK866 on H_2O_2-induced ROS production in HUVEC (A), mBMDM (B), and hDPSCs (C). Cells were pretreated with or without FK866 (10 μM) for 2 h and then stimulated with H_2O_2 (400 nM) for 24 h. Measurement of the total intracellular ROS levels in H_2O_2-treated cells with or without FK866 pretreatment using a fluorescent microplate reader. *$p < 0.1$, **$p < 0.01$.
